# Serum biomarkers identify critically ill traumatic brain injury patients for MRI

**DOI:** 10.1186/s13054-022-04250-3

**Published:** 2022-11-29

**Authors:** Sophie Richter, Stefan Winzeck, Endre Czeiter, Krisztina Amrein, Evgenios N. Kornaropoulos, Jan Verheyden, Gabriela Sugar, Zhihui Yang, Kevin Wang, Andrew I. R. Maas, Ewout Steyerberg, András Büki, Virginia F. J. Newcombe, David K. Menon, the Collaborative European NeuroTrauma Effectiveness Research in Traumatic Brain Injury Magnetic Resonance Imaging (CENTER-TBI MRI) Sub-study Participants and Investigators, Krisztina Amrein, Nada Andelic, Lasse Andreassen, Audny Anke, Philippe Azouvi, Bo‑Michael Bellander, Habib Benali, Andras Buki, Alessio Caccioppola, Emiliana Calappi, Marco Carbonara, Giuseppe Citerio, Hans Clusmann, Mark Coburn, Jonathan Coles, Marta Correia, Endre Czeiter, Véronique De Keyser, Vincent Degos, Bart Depreitere, Live Eikenes, Erzsébet Ezer, Kelly Foks, Shirin Frisvold, Damien Galanaud, Alexandre Ghuysen, Ben Glocker, Asta Haberg, Iain Haitsma, Eirik Helseth, Peter J. Hutchinson, Evgenios Kornaropoulos, Noémi Kovács, Ana Kowark, Steven Laureys, Didier Ledoux, Hester Lingsma, Andrew I. R. Maas, Geoffrey Manley, David K. Menon, Tomas Menovsky, Benoit Misset, Visakh Muraleedharan, Ingeborg Nakken, Virginia Newcombe, Wibeke Nordhøy, József Nyirádi, Fabrizio Ortolano, Paul M. Parizel, Vincent Perlbarg, Paolo Persona, Wilco Peul, Jussi P. Posti, Louis Puybasset, Sophie Richter, Cecilie Roe, Olav Roise, Rolf Rossaint, Sandra Rossi, Daniel Rueckert, Toril Skandsen, Abayomi Sorinola, Emmanuel Stamatakis, Ewout W. Steyerberg, Nino Stocchetti, Riikka Takala, Viktória Tamás, Olli Tenovuo, Zoltán Vámos, Gregory Van der Steen, Wim Van Hecke, Thijs Vande Vyvere, Jan Verheyden, Anne Vik, Victor Volovici, Lars T. Westlye, Guy Williams, Stefan Winzeck, Peter Ylén, Tommaso Zoerle

**Affiliations:** 1 University Division of Anaesthesia, University of Cambridge, Box 93, Addenbrooke’s Hospital, Hills Road, Cambridge, CB2 0QQ UK; 2 Biomedical Image Analysis Group, Department of Computing, Imperial College London, London, UK; 3 Department of Neurosurgery, Medical School, University of Pécs, Pecs, Hungary; 4 Neurotrauma Research Group, Szentágothai Research Centre, University of Pécs, Pecs, Hungary; 5 ELKH-PTE Clinical Neuroscience MR Research Group, University of Pécs, Pecs, Hungary; 6 Diagnostic Radiology, Department of Clinical Sciences, Lund University, Lund, Sweden; 7 Research and Development, icometrix, Leuven, Belgium; 8 Brain and Spinal Injury Center, University of California, San Francisco, San Francisco, CA USA; 9 Program for Neurotrauma, Neuroproteomics and Biomarker Research, Departments of Emergency Medicine, Psychiatry and Neuroscience, University of Florida, Gainesville, USA; 10 Department of Neurosurgery, Antwerp University Hospital and University of Antwerp, Edegem, Belgium; 11 Department of Biomedical Data Sciences, University Medical Centre, Leiden, Netherlands; 12 Department of Neurosurgery, School of Medical Sciences, Faculty of Medicine and Health, Örebro University, Örebro, Sweden

**Keywords:** Traumatic brain injury, Traumatic axonal injury, Diffuse axonal injury, Magnetic resonance imaging, Glasgow Coma Scale, Serum protein biomarkers, Neuron-specific enolase (NSE), Tau, Ubiquitin C terminal hydrolase L1 (UCH-L1), Glial fibrillary acidic protein (GFAP)

## Abstract

**Background:**

Magnetic resonance imaging (MRI) carries prognostic importance after traumatic brain injury (TBI), especially when computed tomography (CT) fails to fully explain the level of unconsciousness. However, in critically ill patients, the risk of deterioration during transfer needs to be balanced against the benefit of detecting prognostically relevant information on MRI. We therefore aimed to assess if day of injury serum protein biomarkers could identify critically ill TBI patients in whom the risks of transfer are compensated by the likelihood of detecting management-altering neuroimaging findings.

**Methods:**

Data were obtained from the Collaborative European NeuroTrauma Effectiveness Research in Traumatic Brain Injury (CENTER-TBI) study. Eligibility criteria included: TBI patients aged ≥ 16 years, Glasgow Coma Score (GCS) < 13 or patient intubated with unrecorded pre-intubation GCS, CT with Marshall score < 3, serum biomarkers (GFAP, NFL, NSE, S100B, Tau, UCH-L1) sampled ≤ 24 h of injury, MRI < 30 days of injury. The degree of axonal injury on MRI was graded using the Adams-Gentry classification. The association between serum concentrations of biomarkers and Adams-Gentry stage was assessed and the optimum threshold concentration identified, assuming different minimum sensitivities for the detection of brainstem injury (Adams-Gentry stage 3). A cost–benefit analysis for the USA and UK health care settings was also performed.

**Results:**

Among 65 included patients (30 moderate-severe, 35 unrecorded) axonal injury was detected in 54 (83%) and brainstem involvement in 33 (51%). In patients with moderate-severe TBI, brainstem injury was associated with higher concentrations of NSE, Tau, UCH-L1 and GFAP. If the clinician did not want to miss any brainstem injury, NSE could have avoided MRI transfers in up to 20% of patients. If a 94% sensitivity was accepted considering potential transfer-related complications, GFAP could have avoided 30% of transfers. There was no added net cost, with savings up to £99 (UK) or $612 (US). No associations between proteins and axonal injury were found in intubated patients without a recorded pre-intubation GCS.

**Conclusions:**

Serum protein biomarkers show potential to safely reduce the number of transfers to MRI in critically ill patients with moderate-severe TBI at no added cost.

**Supplementary Information:**

The online version contains supplementary material available at 10.1186/s13054-022-04250-3.

## Introduction

Traumatic brain injury (TBI) accounts for 300,000 hospitalizations in the US and 1.5 million hospitalization in Europe every year [1]. Some patients present with a low Glasgow Coma Scale score (GCS) [2] without evidence of mass lesion or raised intracranial pressure on computed tomography (CT). The low GCS may be caused either by CT-occult traumatic (diffuse) axonal injury, or by more reversible factors such as alcohol, drugs and/or seizures. Other patients are emergently intubated at the injury scene to manage extra-cranial injuries and arrive at the hospital without a recorded pre-intubation GCS. In these patients, a normal CT does not preclude the presence of CT-occult injury.

Magnetic resonance imaging (MRI) is increasingly used to detect such CT-occult axonal injury. High-grade axonal injury (particularly brainstem injury) is clinically important, since it tends to drive outcome [3, 4]. According to the Adams-Gentry grading, foci confined to the hemispheres indicate stage 1, foci involving the corpus callosum stage 2 and foci in the brainstem stage 3 [5, 6]. Such information is critical when considering interventions such as decompressive craniectomy, which can increase survival in refractory intracranial hypertension, but at the risk of unacceptable disability in patients in whom outcome is driven by the primary injury rather than secondary insults of intracranial hypertension [7].

The prognostic benefit of MRI, however, needs to be weighed against the clinical risk of patient transfer to the scanner, even if MRI is available within the same hospital. Previous studies found that one in four intra-hospital transfers of ventilated patients is associated with complications (1.5% with life-threatening complications), which occur at twice the rate seen in non-transferred patients [8, 9]. A serum biomarker of axonal injury as a triage tool for MRI would thus be useful. Serum protein biomarkers, especially glial fibrillary acidic protein, have been shown to detect CT-occult (axonal) injury in mild TBI, but their utility in moderate-severe TBI is still unclear [10]. To be useful, serum biomarkers would need to prove clinically safe (i.e., reach an acceptable minimum sensitivity for the detection of brainstem injury) and affordable (i.e., not generating large additional costs).

We therefore aimed to investigate if serum protein biomarkers could identify critically ill TBI patients for MRI.

## Methods

Patients were selected from the Collaborative European NeuroTrauma Effectiveness Research in Traumatic Brain Injury (CENTER-TBI) study [11]. Clinical data was accessed via the Neurobot platform (RRID/SCR_017004, core data, version 3.0; International Neuroinformatics Coordinating Facility; released November 24, 2020).

The present analysis included all patients in whom the CT did not fully explain the GCS, defined as:

1. a CT within 24 h of injury without evidence of raised intracranial pressure or mass lesion i.e., Marshall score < 3 [12].
2. PLUS
  - a GCS < 13 (“moderate-severe sub-cohort”) OR
  - a GCS that was unrecorded prior to intubation (“unrecorded sub-cohort”).

In addition, all patients must have been aged ≥ 16 years, undergone MRI within 30 days of injury, and had serum protein biomarkers sampled within 24 h. Glial fibrillary acidic protein (GFAP), neurofilament light (NFL), neuron-specific enolase (NSE), S100 calcium-binding protein B (S100B), total tau (Tau) and ubiquitin carboxy-terminal hydrolase L1 (UCH-L1) were assayed as described previously [13].

### Image acquisition, and reporting

CT images were acquired according to local site protocols, were reported centrally by trained investigators blinded to outcome and assigned a Marshall score [12, 14].

MR images were obtained following study-specific protocols (https://www.center-tbi.eu/project/mri-study-protocols) and included T1-weighted, T2-weighted, fluid-attenuated inversion recovery, susceptibility-weighted and diffusion-weighted images. The location of axonal injury on MRI was reported in Cambridge by one neurotrauma research clinician blinded to patient characteristics (SR) and reviewed by a second neurotrauma research clinician (VFJN). The degree of axonal injury was scored using the Adams-Gentry classification [5, 6]. For axonal brainstem injury we recorded if known adverse features were present, i.e., injury that was bilateral, dorsal, pontine or associated with Duret hemorrhage or contusion [4, 15, 16].

### Statistical analysis

The associations between proteins and the Adams-Gentry stage or the presence of brainstem injury was assessed with a two-sided Jonckheere-Terpstra test and a Mann–Whitney U test, respectively. Statistical analysis was conducted in R 4.2.0 (R Project for Statistical Computing). The significance threshold for p values was set at 0.05 and all p values adjusted for multiple comparisons using the Benjamini–Hochberg method [17].

For each protein we identified the serum concentration where specificity would be maximal given a minimum sensitivity of (a) 90% or (b) 100%, using the R package OptimalCutpoints. The cost–benefit analysis was conducted with the perspectives of the UK and the USA health care systems (Additional file 1: Methods).

## Results

Inclusion criteria were met by 65 patients (30 in the moderate-severe sub-cohort, 35 in the unrecorded sub-cohort), of which 49 (75%) were male, 60 (92%) were intubated and 39 (60%) had sustained major extra-cranial injuries; the median age was 40 years (range 16–82) (Additional file 1: Table S1). The study population was younger and more severely injured than the whole CENTER-TBI population (Additional file 1: Table S2). The MRI was performed at a median of 6 days (range 0–29), showed axonal injury in 54 (83%) and brainstem involvement 33 (51%) patients (Additional file 1: Table S3).

Axonal injury burden irrespective of location was associated with serum GFAP concentrations (Additional file 1: Fig. S1). The Adams-Gentry stage was associated with GFAP, NSE and UCH-L1 in the moderate-severe sub-cohort but was not associated with any proteins in the unrecorded sub-cohort or the overall study cohort (Additional file 1: Table S4).

Adams-Gentry stage 3 (brainstem involvement) was associated with GFAP (borderline statistical significance), NSE, Tau and UCH-L1 in the moderate-severe sub-cohort but was not associated with any proteins in the unrecorded sub-cohort or the overall study cohort (Table 1).

**Table 1:** Association between serum protein concentration and brainstem injury (Adams-Gentry stage 3)

	Brainstem injury absent	Brainstem injury present	Adj. p value
*Overall cohort*			
Number of patients	32 (49%)	33 (51%)	
Sample time in hours	16 (11–21)	17 (10–18)	0.600
GFAP	10.19 (4.03–28.13)	27.07 (9.41–38.60)	0.184
NFL	28.13 (20.82–102.72)	43.82 (28.86–82.09)	0.600
NSE	20.33 (15.30–33.97)	23.58 (20.31–39.52)	0.184
S100B	0.29 (0.17–0.66)	0.27 (0.20–0.51)	1.000
Tau	5.55 (3.06–12.09)	8.90 (3.33–17.07)	0.600
UCH-L1	237.28 (108.64–526.42)	466.39 (169.42–899.86)	0.184
*Sub-cohort with moderate-severe TBI*			
Number of patients	13 (43%)	17 (57%)	
Sample time in hours	14 (10–21)	16 (10–20)	0.711
GFAP	4.46 (3.11–25.91)	30.31 (18.90–40.99)	**0.050**
NFL	27.82 (18.63–47.75)	47.21 (37.55–81.19)	0.186
NSE	17.14 (13.38–25.05)	31.91 (22.98–46.36)	**0.041**
S100B	0.17 (0.07–0.24)	0.29 (0.22–0.51)	0.093
Tau	4.31 (2.17–7.27)	14.36 (4.47–21.49)	**0.046**
UCH-L1	123.48 (98.13–262.49)	696.42 (218.56–953.05)	**0.040**
*Sub-cohort with unrecorded GCS*			
Number of patients	19 (54%)	16 (46%)	
Sample time in hours	16 (11–21)	17 (10–18)	0.803
GFAP	12.69 (5.21–28.57)	21.84 (9.39–33.22)	0.803
NFL	28.45 (21.43–125.89)	35.74 (18.39–83.29)	0.878
NSE	20.90 (18.31–34.00)	22.84 (19.34–26.29)	0.987
S100B	0.46 (0.22–0.66)	0.25 (0.16–0.47)	0.512
Tau	9.01 (4.76–13.92)	4.81 (2.94–11.34)	0.803
UCH-L1	379.55 (178.36–557.11)	273.19 (128.58–503.10)	0.936

Values are presented as count (percent) or median (first quartile – third quartile). Serum protein concentrations are measured in ng/ml for GFAP, NSE and S100B, and in pg/ml for NFL, Tau and UCH-L1. Adj. *p* value = *P* values from the Mann–Whitney *U*-test adjusted for multiple comparisons. Significant p values are in bold. TBI = traumatic brain injury, GCS = Glasgow Coma Scale score

In the moderate-severe sub-cohort protein biomarkers showed potential for MRI triage. Assuming a minimum sensitivity of 90% for the detection of brainstem injury, the best specificity was achieved by GFAP, avoiding 30% of MRI transfers whilst missing 1 in 20 brainstem injuries (Table 2). If a sensitivity of 100% was desired, then NSE performed best, avoiding 20% of MRI transfers whilst not missing any brainstem injury (Table 2 and Additional file 1: Table S5). Both approaches were cost-saving.

**Table 2:** Using serum protein biomarkers for MRI triage in patients with moderate-severe traumatic brain injury

Protein	Threshold	Sensitivity	Specificity	Patients above threshold	Costs in the United Kingdom (GBP per patient)			Costs in the United States (USD per patient)		
					MRI for all	Protein plus selected MRI	Savings	MRI for all	Protein plus selected MRI	Savings
GFAP	5.73	0.94	0.62	21 (70%)	385.80	286.88	98.92	2758.56	1996.15	762.41
NFL	19.42	0.94	0.38	24 (80%)	385.80	331.09	54.71	2758.56	–	–
NSE	16.61	1.00	0.46	24 (80%)	385.80	322.32	63.48	2758.56	–	–
S100B	0.09	0.94	0.38	24 (80%)	385.80	330.20	55.60	2758.56	–	–
Tau	2.34	1.00	0.31	26 (87%)	385.80	355.42	30.38	2758.56	–	–
UCH-L1	133.23	0.94	0.54	22 (73%)	385.80	298.95	86.85	2758.56	2088.10	670.46

*N* = 30. Threshold = optimal cut-off for the serum protein concentration measured in ng/ml for GFAP, NSE and S100B, and in pg/ml for NFL, Tau and UCH-L1. “Optimal” means maximizing the specificity whilst achieving a minimum sensitivity of 0.90. MRI for all = cost per patient of subjecting all patients to magnetic resonance imaging (MRI). Protein plus selected MRI = cost per patient of sampling serum protein biomarkers from all patients and taking only those patients for MRI who exceed the threshold serum concentration. Savings = Cost savings when using Protein plus selected MRI compared to MRI for all. To gain an estimate of real-world clinical costs, the United States costs are not based on the assays used in the present study (which are for research only) but on the FDA-approved i-stat platform by Abbott. This platform only measures GFAP and UCH-L1, so costs for other proteins were not calculated

The prevalence of adverse features of brainstem injury is documented in Additional file 1: Table S3. In the moderate-severe sub-cohort, GFAP, NSE and UCH-L1 were associated with adverse features (Additional file 1: Table S6).

## Discussion

This study evaluated whether protein biomarkers could help avoid high-risk clinical transfers for MRI in critically ill TBI patients in whom the CT may not fully explain the GCS.

The risk of deterioration during transfer varies widely depending on, for example, the patient’s clinical status, the availability of trained personnel and the distance to the MRI scanner [8, 9]. Depending on this estimated risk a clinician may tolerate different levels of sensitivity for protein biomarkers. We identified NSE as the most promising biomarker in patients at lower risk of transfer-related complications and GFAP as the most promising biomarker when transfer-related adverse events are likely. In both scenarios, protein biomarkers were not only affordable but cost-saving.

We found GFAP, NSE, UCH-L1 outperformed axonal markers (NFL and Tau) for the detection of axonal injury. Sampling within 24 h may have discriminated against the slower to peak NFL. Protein biomarker concentrations have previously been found using CT to reflect the total burden of injury irrespective of lesion type or location [18]. The complex pathophysiology after TBI including traumatic vascular injury, blood brain barrier disruption and a host inflammatory response means biomarker elevations may not only result from axonal injury.

We considered whether the poor relationship between biomarkers and MRI in the unrecorded sub-cohort might be due to a lower severity of brain stem injury in these emergently intubated patients, which however was not the case. Despite additional exploration of the data, we were unable to find a satisfactory explanation. Possible confounds that we are unable to test for in our data include high volume transfusions which may have diluted biomarker levels; second insults not reflected in admission biomarker levels; or a Type II error due to relatively small sample sizes in subgroups, which may be addressed in a larger study.

## Limitations

The sample size of 65, while large for a prospective study of early MRI after moderate-to-severe TBI, requires external validation in larger cohorts. This would also enable more refined analysis of the influence of lesion location and type. Furthermore, Quanterix assay kits are currently only available for research purposes. However, an alternative platform is already licensed for GFAP and UCH-L1 and more are likely to be approved in the future [19]. Our time window for MRI ranged from 0 to 30 days. As imaging features change during this timeframe, future studies should aim for a more uniform imaging timepoint [4, 20]. Our findings should therefore not be interpreted as a call to change clinical practice, but as an encouragement to repeat this study in a larger cohort.

## Conclusion

Serum protein biomarkers show promise as a triage tool for MRI in TBI patients where the CT does not fully explain a low GCS, but not in patients with an unrecorded GCS. Findings require validation in a larger cohort.

## Supplementary Information

**Additional file 1.** Supplemental methods, figures and tables.

## Data Availability

CENTER-TBI investigators are strong proponents of data sharing to advance TBI research. De-identified patient data is available upon request, subject to approval by the CENTER-TBI Management Committee. Proposals can be submitted online at https://www.center-tbi.eu/data and will be assessed for methodological soundness. Shared data can be used without a time limit but in the context of a data sharing agreement and in accordance with the regulatory restrictions of the original CENTER-TBI study. The statistical analysis code is freely available at https://github.com/DrSophieRichter/MRI_triage.

## Abbreviations

TBI: Traumatic brain injury
CT: Computed tomography
MRI: Magnetic resonance imaging
GFAP: Glial fibrillary acidic protein
NFL: Neurofilament light
NSE: Neuron-specific enolase
S100B: Calcium-binding protein B
Tau: Total tau
UCH-L1: Ubiquitin carboxy-terminal hydrolase L1
