# Supplementary material for: Serum biomarkers identify critically ill traumatic brain injury patients for MRI
Source: Crit Care. 2022 Nov 29;26:369. doi: 10.1186/s13054-022-04250-3 (PMC9706877; doi:10.1186/s13054-022-04250-3)
Supplement: Supplementary file 1 — Additional file 1. Supplemental methods, figures and tables. [file 13054_2022_4250_MOESM1_ESM.docx]

Supplement to

“Serum biomarkers identify critically ill traumatic brain injury patients for MRI”

**Authors**

Sophie Richter, MD; Stefan Winzeck, PhD; Endre Czeiter, MD PhD; Krisztina Amrein, MSc; Evgenios N Kornaropoulos, PhD; Jan Verheyden, MSc; Gabriela Sugar; Zhihui Yang, PhD; Kevin Wang, PhD; Andrew I R Maas, MD PhD; Ewout Steyerberg, PhD; András Büki, MD PhD; Virginia F J Newcombe, MD PhD; David K Menon, MD PhD and the Collaborative European NeuroTrauma Effectiveness Research in Traumatic Brain Injury Magnetic Resonance Imaging (CENTER-TBI MRI) Sub-study Participants and Investigators

# Contents

1. Supplemental methods. Cost-benefit analysis
2. Supplemental table 1. Patient characteristics
3. Supplemental table 2. Comparison of present study cohort with whole CENTER-TBI study population
4. Supplemental table 3. Imaging findings
5. Supplemental figure 1. Association of serum protein concentration with axonal injury burden
6. Supplemental table 4. Association between serum protein concentration and Adams-Gentry stage
7. Supplemental table 5: Using serum protein biomarkers for MRI triage in patients with moderate-severe traumatic brain injury.
8. Supplemental table 6. Association of protein concentration with adverse features of axonal brainstem injury in the moderate-severe sub-cohort.
9. References

# Supplemental methods: Cost-benefit analysis

The cost-benefit analysis was performed using a health care perspective and considering only direct medical costs. We compared the cost of testing all patients with protein biomarkers and then scanning only those with concentrations above the threshold versus the cost of scanning all patients without sampling any protein biomarkers. As costs differ widely across health care systems, we conducted the analysis separately using costs for the English National Health Service (NHSE) and for the United States of America (USA) health care system. For USA context, we assessed the benefit of GFAP and UCH-L1 only as these were the two proteins with the highest specificity and the only proteins for which an assay platform is already available in routine clinical practice.

For the context of the NHSE, the cost of an MRI was estimated as the combined cost of a double session on the MRI machine (to take into account the extra time required to transfer and scan a ventilated patient) as well as an appropriately trained doctor (for example an anesthetist in training) and a nurse to accompany the patient for two hours. The National Schedule of NHS costs 2019/2020 states the cost of a magnetic resonance scan of one area without contrast in a patient 19 years and over (RD01A) as £146.75.^1^ In England the annual salary of a junior anaesthetist (ST3-ST5 level, resident or registrar equivalent) working 40 hours per week, of which 10 out of hours, is £55,736 annually or £26.80 per hour.^2^ The hourly salary for an appropriate level nurse (band 5) is £19.35.^3^ This makes the cost of one MRI head in our cohort to be approximately £385.80. The cost of a serum protein biomarker test was estimated as the cost of phlebotomy (venepuncture, lab technician etc.) plus the cost of the specialised assay kit. In the NHSE phlebotomy (DAPS08) costs on average £4.^1^ The per test cost for Quanterix assay kits are as follows: GFAP £12.82, NFL £18.45, Tau £17.06 and UCH-L1 £12.03 (personal communication with Ellen Hegarty, Inside Sales Representative UK, Ireland & Nordics, Quanterix). The per test cost for Roche assay kits are £9.68 for NSE and £17.56 for S100B (personal communication with Dr Joy Allen, Health Economics Manager, Access & Innovation, Roche Diagnostics Limited).

For the context of the USA health care system, the cost of an MRI was taken as the reported national average ($2,625) plus the cost of a medical resident ($27 per hour) and an appropriately trained nurse ($39.78 per hour) to accompany the patient for two hours, yielding a total of $2758.56 per MRI.^4-6^ The cost of testing for GFAP and UCH-L1 using the clinically available i-stat platform by Abbott was estimated as the cost of phlebotomy ($15.16, personal communication with Gabriela Sugar, research nurse, University of California San Francisco) plus the cost of the i-stat assay kits per patient ($50, personal communication with Mark Powell, UK & Ireland Country Manager APOC, Abbott Laboratories Ltd).

|  | Sub-cohort with moderate-severe TBI (N=30) | Sub-cohort with unrecorded GCS (N=35) | Overall (N=65) |
| --- | --- | --- | --- |
| **Age** |  |  |  |
| Median (Min-Max) | 38 (17 - 70) | 41 (16 - 82) | 40 (16 - 82) |
| **Sex** |  |  |  |
| F | 7 (23 %) | 9 (26 %) | 16 (25 %) |
| M | 23 (77 %) | 26 (74 %) | 49 (75 %) |
| **Pre-injury ASA grade** |  |  |  |
| 1 | 23 (77 %) | 19 (54 %) | 42 (65 %) |
| 2 | 6 (20 %) | 11 (31 %) | 17 (26 %) |
| 3 | 1 (3 %) | 5 (14 %) | 6 (9 %) |
| 4 | 0 (0 %) | 0 (0 %) | 0 (0 %) |
| **Prior neurological disease** |  |  |  |
| absent | 29 (97 %) | 34 (97 %) | 63 (97 %) |
| present | 1 (3 %) | 1 (3 %) | 2 (3 %) |
| **Prior psychiatric disease** |  |  |  |
| absent | 22 (73 %) | 31 (89 %) | 53 (82 %) |
| present | 5 (17 %) | 4 (11 %) | 9 (14 %) |
| Missing | 3 (10.0%) | 0 (0%) | 3 (4.6%) |
| **Prior renal disease** |  |  |  |
| absent | 27 (90 %) | 34 (97 %) | 61 (94 %) |
| present | 2 (7 %) | 1 (3 %) | 3 (5 %) |
| Missing | 1 (3.3%) | 0 (0%) | 1 (1.5%) |
| **Prior hepatic disease** |  |  |  |
| absent | 30 (100 %) | 34 (97 %) | 64 (98 %) |
| present | 0 (0 %) | 1 (3 %) | 1 (2 %) |
| **Admission status** |  |  |  |
| ward | 1 (3 %) | 0 (0 %) | 1 (2 %) |
| ICU (self-ventilating) | 2 (7 %) | 0 (0 %) | 2 (3 %) |
| ICU (intubated) | 25 (83 %) | 35 (100 %) | 60 (92 %) |
| ICU (airway unknown) | 2 (7 %) | 0 (0 %) | 2 (3 %) |
| **Major extra-cranial injury** |  |  |  |
| absent | 12 (40 %) | 14 (40 %) | 26 (40 %) |
| present | 18 (60 %) | 21 (60 %) | 39 (60 %) |
| **Number of major extracranial injuries** |  |  |  |
| Median (Min-Max) | 1.0 (0 - 6) | 1.0 (0 - 6) | 1.0 (0 - 6) |
| **Major head/neck injury** |  |  |  |
| absent | 14 (47 %) | 14 (40 %) | 28 (43 %) |
| present | 16 (53 %) | 21 (60 %) | 37 (57 %) |
| **Major face injury** |  |  |  |
| absent | 21 (70 %) | 24 (69 %) | 45 (69 %) |
| present | 9 (30 %) | 11 (31 %) | 20 (31 %) |
| **Major chest injury** |  |  |  |
| absent | 14 (47 %) | 19 (54 %) | 33 (51 %) |
| present | 16 (53 %) | 16 (46 %) | 32 (49 %) |
| **Major thoracic spine injury** |  |  |  |
| absent | 27 (90 %) | 35 (100 %) | 62 (95 %) |
| present | 3 (10 %) | 0 (0 %) | 3 (5 %) |
| **Major lumbar spine injury** |  |  |  |
| absent | 28 (93 %) | 33 (94 %) | 61 (94 %) |
| present | 2 (7 %) | 2 (6 %) | 4 (6 %) |
| **Major abdomen/pelvis injury** |  |  |  |
| absent | 27 (90 %) | 30 (86 %) | 57 (88 %) |
| present | 3 (10 %) | 5 (14 %) | 8 (12 %) |
| **Major upper extremity injury** |  |  |  |
| absent | 27 (90 %) | 31 (89 %) | 58 (89 %) |
| present | 3 (10 %) | 4 (11 %) | 7 (11 %) |
| **Major lower extremity injury** |  |  |  |
| absent | 22 (73 %) | 29 (83 %) | 51 (78 %) |
| present | 8 (27 %) | 6 (17 %) | 14 (22 %) |
| **Major external injury** |  |  |  |
| absent | 29 (97 %) | 33 (94 %) | 62 (95 %) |
| present | 1 (3 %) | 2 (6 %) | 3 (5 %) |
| **Glasgow Coma Score** |  |  |  |
| unrecorded | 0 (0 %) | 35 (100 %) | 35 (54 %) |
| 3 | 9 (30 %) | 0 (0 %) | 9 (14 %) |
| 4 | 3 (10 %) | 0 (0 %) | 3 (5 %) |
| 5 | 2 (7 %) | 0 (0 %) | 2 (3 %) |
| 6 | 2 (7 %) | 0 (0 %) | 2 (3 %) |
| 7 | 4 (13 %) | 0 (0 %) | 4 (6 %) |
| 8 | 1 (3 %) | 0 (0 %) | 1 (2 %) |
| 9 | 1 (3 %) | 0 (0 %) | 1 (2 %) |
| 10 | 4 (13 %) | 0 (0 %) | 4 (6 %) |
| 11 | 3 (10 %) | 0 (0 %) | 3 (5 %) |
| 12 | 1 (3 %) | 0 (0 %) | 1 (2 %) |
| **Unreactive pupils** |  |  |  |
| 0 | 23 (77 %) | 28 (80 %) | 51 (78 %) |
| 1 | 2 (7 %) | 4 (11 %) | 6 (9 %) |
| 2 | 4 (13 %) | 3 (9 %) | 7 (11 %) |
| Missing | 1 (3.3%) | 0 (0%) | 1 (1.5%) |
| **Early seizure** |  |  |  |
| none | 23 (77 %) | 32 (91 %) | 55 (85 %) |
| partial | 0 (0 %) | 0 (0 %) | 0 (0 %) |
| generalised | 2 (7 %) | 1 (3 %) | 3 (5 %) |
| status epilepticus | 1 (3 %) | 0 (0 %) | 1 (2 %) |
| Missing | 4 (13.3%) | 2 (5.7%) | 6 (9.2%) |
| **Hypoxia** |  |  |  |
| absent | 18 (60 %) | 23 (66 %) | 41 (63 %) |
| suspected | 4 (13 %) | 5 (14 %) | 9 (14 %) |
| present | 7 (23 %) | 3 (9 %) | 10 (15 %) |
| Missing | 1 (3.3%) | 4 (11.4%) | 5 (7.7%) |
| **Hypotension** |  |  |  |
| absent | 25 (83 %) | 26 (74 %) | 51 (78 %) |
| suspected | 0 (0 %) | 0 (0 %) | 0 (0 %) |
| present | 4 (13 %) | 7 (20 %) | 11 (17 %) |
| Missing | 1 (3.3%) | 2 (5.7%) | 3 (4.6%) |
| **Alcohol intoxication** |  |  |  |
| absent | 17 (57 %) | 26 (74 %) | 43 (66 %) |
| suspected | 1 (3 %) | 2 (6 %) | 3 (5 %) |
| present | 6 (20 %) | 5 (14 %) | 11 (17 %) |
| Missing | 6 (20.0%) | 2 (5.7%) | 8 (12.3%) |
| **Time to blood protein sample (hours)** |  |  |  |
| Median (Min-Max) | 15 (2.2 - 23) | 17 (3 - 23.2) | 16 (2.2 - 23.2) |
| **Marshall score** |  |  |  |
| 1 | 2 (7 %) | 6 (17 %) | 8 (12 %) |
| 2 | 24 (80 %) | 27 (77 %) | 51 (78 %) |
| MRI equivalent to 1 or 2 | 4 (13 %) | 2 (6 %) | 6 (9 %) |
| **CT - TAI** |  |  |  |
| absent | 17 (57 %) | 20 (57 %) | 37 (57 %) |
| present | 8 (27 %) | 11 (31 %) | 19 (29 %) |
| Missing | 5 (16.7%) | 4 (11.4%) | 9 (13.8%) |
| **CT - midline shift** |  |  |  |
| absent | 24 (80 %) | 31 (89 %) | 55 (85 %) |
| present | 1 (3 %) | 0 (0 %) | 1 (2 %) |
| Missing | 5 (16.7%) | 4 (11.4%) | 9 (13.8%) |
| **CT - cisternal compression** |  |  |  |
| absent | 24 (80 %) | 31 (89 %) | 55 (85 %) |
| present | 1 (3 %) | 0 (0 %) | 1 (2 %) |
| Missing | 5 (16.7%) | 4 (11.4%) | 9 (13.8%) |
| **CT - SAH** |  |  |  |
| absent | 8 (27 %) | 10 (29 %) | 18 (28 %) |
| present | 17 (57 %) | 21 (60 %) | 38 (58 %) |
| Missing | 5 (16.7%) | 4 (11.4%) | 9 (13.8%) |
| **CT - EDH** |  |  |  |
| absent | 23 (77 %) | 27 (77 %) | 50 (77 %) |
| present | 2 (7 %) | 4 (11 %) | 6 (9 %) |
| Missing | 5 (16.7%) | 4 (11.4%) | 9 (13.8%) |
| **CT - haematoma** |  |  |  |
| absent | 21 (70 %) | 21 (60 %) | 42 (65 %) |
| present | 4 (13 %) | 10 (29 %) | 14 (22 %) |
| Missing | 5 (16.7%) | 4 (11.4%) | 9 (13.8%) |
| **Any neurosurgery** |  |  |  |
| not performed | 22 (73 %) | 28 (80 %) | 50 (77 %) |
| performed | 7 (23 %) | 7 (20 %) | 14 (22 %) |
| Missing | 1 (3.3%) | 0 (0%) | 1 (1.5%) |
| **Emergency neurosurgery** |  |  |  |
| not performed | 27 (90 %) | 31 (89 %) | 58 (89 %) |
| performed | 2 (7 %) | 4 (11 %) | 6 (9 %) |
| Missing | 1 (3.3%) | 0 (0%) | 1 (1.5%) |
| **Decompressive craniectomy** |  |  |  |
| not performed | 28 (93 %) | 33 (94 %) | 61 (94 %) |
| performed | 1 (3 %) | 2 (6 %) | 3 (5 %) |
| Missing | 1 (3.3%) | 0 (0%) | 1 (1.5%) |
| **GOSE at 6 months** |  |  |  |
| 1 | 0 (0 %) | 4 (11 %) | 4 (6 %) |
| 2_or_3 | 6 (20 %) | 5 (14 %) | 11 (17 %) |
| 4 | 4 (13 %) | 4 (11 %) | 8 (12 %) |
| 5 | 5 (17 %) | 5 (14 %) | 10 (15 %) |
| 6 | 7 (23 %) | 5 (14 %) | 12 (18 %) |
| 7 | 3 (10 %) | 4 (11 %) | 7 (11 %) |
| 8 | 3 (10 %) | 2 (6 %) | 5 (8 %) |
| Missing | 2 (6.7%) | 6 (17.1%) | 8 (12.3%) |

***Supplemental table 1. Patient characteristics.*** *GCS = Glasgow Coma Scale score, ICU = intensive care unit, CT = computed tomography, SAH = subarachnoid haemorrhage, EDH = extradural haemorrhage. Note that neurosurgery includes insertion of intra-cranial pressure monitoring devices and intra-ventricular drains.*

|  | included (N=65) | not included (N=4444) | Overall (N=4509) |
| --- | --- | --- | --- |
| **Age** |  |  |  |
| Median (Min-Max) | 40 (16 - 82) | 50 (0 - 96) | 50 (0 - 96) |
| Missing | 0 (0%) | 14 (0.3%) | 14 (0.3%) |
| **Sex** |  |  |  |
| F | 16 (25 %) | 1470 (33 %) | 1486 (33 %) |
| M | 49 (75 %) | 2974 (67 %) | 3023 (67 %) |
| **Admission status** |  |  |  |
| not admitted | 0 (0 %) | 848 (19 %) | 848 (19 %) |
| ward | 1 (2 %) | 1522 (34 %) | 1523 (34 %) |
| ICU (self-ventilating) | 2 (3 %) | 546 (12 %) | 548 (12 %) |
| ICU (intubated) | 60 (92 %) | 1502 (34 %) | 1562 (35 %) |
| ICU (airway unknown) | 2 (3 %) | 26 (1 %) | 28 (1 %) |
| **Major extra-cranial injury** |  |  |  |
| absent | 26 (40 %) | 3217 (72 %) | 3243 (72 %) |
| present | 39 (60 %) | 1227 (28 %) | 1266 (28 %) |
| **Glasgow Coma Scale** |  |  |  |
| 3 | 9 (14 %) | 336 (8 %) | 345 (8 %) |
| 4 | 3 (5 %) | 38 (1 %) | 41 (1 %) |
| 5 | 2 (3 %) | 34 (1 %) | 36 (1 %) |
| 6 | 2 (3 %) | 38 (1 %) | 40 (1 %) |
| 7 | 4 (6 %) | 55 (1 %) | 59 (1 %) |
| 8 | 1 (2 %) | 57 (1 %) | 58 (1 %) |
| 9 | 1 (2 %) | 48 (1 %) | 49 (1 %) |
| 10 | 4 (6 %) | 72 (2 %) | 76 (2 %) |
| 11 | 3 (5 %) | 68 (2 %) | 71 (2 %) |
| 12 | 1 (2 %) | 73 (2 %) | 74 (2 %) |
| 13 | 0 (0 %) | 169 (4 %) | 169 (4 %) |
| 14 | 0 (0 %) | 474 (11 %) | 474 (11 %) |
| 15 | 0 (0 %) | 2213 (50 %) | 2213 (49 %) |
| Missing | 35 (53.8%) | 769 (17.3%) | 804 (17.8%) |
| **Unreactive pupils** |  |  |  |
| 0 | 51 (78 %) | 3751 (84 %) | 3802 (84 %) |
| 1 | 6 (9 %) | 158 (4 %) | 164 (4 %) |
| 2 | 7 (11 %) | 274 (6 %) | 281 (6 %) |
| Missing | 1 (1.5%) | 261 (5.9%) | 262 (5.8%) |
| **Hypoxia** |  |  |  |
| absent | 41 (63 %) | 3916 (88 %) | 3957 (88 %) |
| suspected | 9 (14 %) | 89 (2 %) | 98 (2 %) |
| present | 10 (15 %) | 191 (4 %) | 201 (4 %) |
| Missing | 5 (7.7%) | 248 (5.6%) | 253 (5.6%) |
| **Hypotension** |  |  |  |
| absent | 51 (78 %) | 3948 (89 %) | 3999 (89 %) |
| suspected | 0 (0 %) | 71 (2 %) | 71 (2 %) |
| present | 11 (17 %) | 215 (5 %) | 226 (5 %) |
| Missing | 3 (4.6%) | 210 (4.7%) | 213 (4.7%) |
| **Alcohol intoxication** |  |  |  |
| absent | 43 (66 %) | 3080 (69 %) | 3123 (69 %) |
| suspected | 3 (5 %) | 348 (8 %) | 351 (8 %) |
| present | 11 (17 %) | 676 (15 %) | 687 (15 %) |
| Missing | 8 (12.3%) | 340 (7.7%) | 348 (7.7%) |
| **Time to blood protein sample (hours)** |  |  |  |
| Median (Min-Max) | 16 (2.2 - 23.2) | 15 (0.5 - 11872.4) | 15 (0.5 - 11872.4) |
| Missing | 0 (0%) | 763 (17.2%) | 763 (16.9%) |
| **Marshall CT score** |  |  |  |
| 1 | 8 (12 %) | 1662 (37 %) | 1670 (37 %) |
| 2 | 51 (78 %) | 1522 (34 %) | 1573 (35 %) |
| unknown | 6 (9 %) | 311 (7 %) | 317 (7 %) |
| 3 | 0 (0 %) | 172 (4 %) | 172 (4 %) |
| 4 | 0 (0 %) | 34 (1 %) | 34 (1 %) |
| 5 | 0 (0 %) | 35 (1 %) | 35 (1 %) |
| 6 | 0 (0 %) | 708 (16 %) | 708 (16 %) |
| **CT – axonal injury** |  |  |  |
| absent | 37 (57 %) | 3679 (83 %) | 3716 (82 %) |
| present | 19 (29 %) | 353 (8 %) | 372 (8 %) |
| Missing | 9 (13.8%) | 412 (9.3%) | 421 (9.3%) |
| **CT - midline shift** |  |  |  |
| absent | 55 (85 %) | 3563 (80 %) | 3618 (80 %) |
| present | 1 (2 %) | 469 (11 %) | 470 (10 %) |
| Missing | 9 (13.8%) | 412 (9.3%) | 421 (9.3%) |
| **CT - cisternal compression** |  |  |  |
| absent | 55 (85 %) | 3381 (76 %) | 3436 (76 %) |
| present | 1 (2 %) | 651 (15 %) | 652 (14 %) |
| Missing | 9 (13.8%) | 412 (9.3%) | 421 (9.3%) |
| **CT - SAH** |  |  |  |
| absent | 18 (28 %) | 2219 (50 %) | 2237 (50 %) |
| present | 38 (58 %) | 1813 (41 %) | 1851 (41 %) |
| Missing | 9 (13.8%) | 412 (9.3%) | 421 (9.3%) |
| **CT - EDH** |  |  |  |
| absent | 50 (77 %) | 3577 (80 %) | 3627 (80 %) |
| present | 6 (9 %) | 455 (10 %) | 461 (10 %) |
| Missing | 9 (13.8%) | 412 (9.3%) | 421 (9.3%) |
| **CT - haematoma** |  |  |  |
| absent | 42 (65 %) | 2511 (57 %) | 2553 (57 %) |
| present | 14 (22 %) | 1521 (34 %) | 1535 (34 %) |
| Missing | 9 (13.8%) | 412 (9.3%) | 421 (9.3%) |
| **GOSE at 6 months** |  |  |  |
| 1 | 4 (6 %) | 469 (11 %) | 473 (10 %) |
| 2_or_3 | 11 (17 %) | 309 (7 %) | 320 (7 %) |
| 4 | 8 (12 %) | 170 (4 %) | 178 (4 %) |
| 5 | 10 (15 %) | 345 (8 %) | 355 (8 %) |
| 6 | 12 (18 %) | 402 (9 %) | 414 (9 %) |
| 7 | 7 (11 %) | 676 (15 %) | 683 (15 %) |
| 8 | 5 (8 %) | 1383 (31 %) | 1388 (31 %) |
| Missing | 8 (12.3%) | 690 (15.5%) | 698 (15.5%) |

***Supplemental table 2. Comparison of present study cohort with whole CENTER-TBI study population.*** *CT = computed tomography,* *SAH = subarachnoid haemorrhage, EDH = extradural haemorrhage, GOSE = extended Glasgow Outcome Scale score*

|  | Sub-cohort with moderate-severe TBI (N=30) | Sub-cohort with unrecorded GCS (N=35) | Overall (N=65) |
| --- | --- | --- | --- |
| **Time to MRI (days)** |  |  |  |
| Median (Min-Max) | 5.5 (1 - 29) | 7.0 (0 - 27) | 6.0 (0 - 29) |
| **Adams-Gentry stage** |  |  |  |
| 0 | 6 (20 %) | 5 (14 %) | 11 (17 %) |
| 1 | 3 (10 %) | 8 (23 %) | 11 (17 %) |
| 2 | 4 (13 %) | 6 (17 %) | 10 (15 %) |
| 3 | 17 (57 %) | 16 (46 %) | 33 (51 %) |
| **Brainstem injury - any type** |  |  |  |
| absent | 13 (43 %) | 19 (54 %) | 32 (49 %) |
| present | 17 (57 %) | 16 (46 %) | 33 (51 %) |
| **Brainstem injury - bilateral** |  |  |  |
| grade <3 | 13 (43 %) | 19 (54 %) | 32 (49 %) |
| absent | 10 (33 %) | 9 (26 %) | 19 (29 %) |
| present | 7 (23 %) | 7 (20 %) | 14 (22 %) |
| **Brainstem injury - dorsal** |  |  |  |
| grade <3 | 13 (43 %) | 19 (54 %) | 32 (49 %) |
| absent | 9 (30 %) | 8 (23 %) | 17 (26 %) |
| present | 8 (27 %) | 8 (23 %) | 16 (25 %) |
| **Brainstem injury - pontine** |  |  |  |
| grade <3 | 13 (43 %) | 19 (54 %) | 32 (49 %) |
| absent | 12 (40 %) | 12 (34 %) | 24 (37 %) |
| present | 5 (17 %) | 4 (11 %) | 9 (14 %) |
| **Brainstem injury - contusion or Duret haemorrhage** |  |  |  |
| grade <3 | 13 (43 %) | 19 (54 %) | 32 (49 %) |
| absent | 14 (47 %) | 14 (40 %) | 28 (43 %) |
| present | 3 (10 %) | 2 (6 %) | 5 (8 %) |

***Supplemental table 3. Imaging findings.*** *MRI = magnetic resonance imaging*


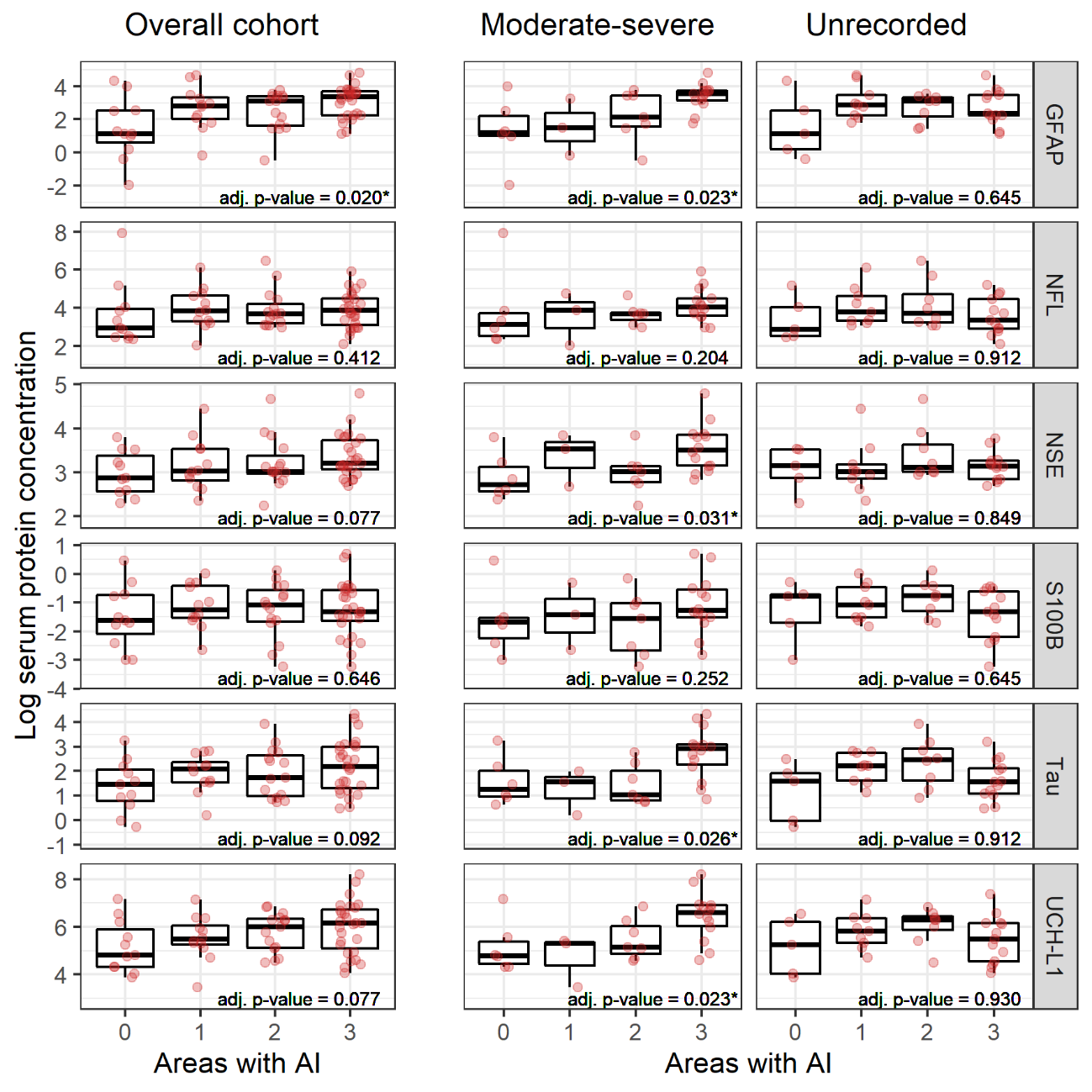


***Supplemental figure 1. Association of serum protein concentration with axonal injury burden****. Areas with AI = The number of brain areas with axonal injury on magnetic resonance imaging. Areas considered were those contained in the Adams-Gentry classification, i.e., hemispheres, corpus callosum and brainstem. Boxplots show the median and interquartile range, red dots are the raw data values for serum biomarker concentrations (log-transformed). P-values were adjusted for multiple comparisons using the false discovery rate and significant p-values highlighted with an asterisk.*

|  | Stage 0 | Stage 1 | Stage 2 | Stage 3 | Adj. p-value |
| --- | --- | --- | --- | --- | --- |
| **Overall** |  |  |  |  |  |
| Number of patients | 11 (17%) | 11 (17%) | 10 (15%) | 33 (51%) |  |
| Sample time in hours | 12 (10-19) | 17 (13-20) | 18 (10-22) | 17 (10-18) | 0.639 |
| GFAP | 3.11  (1.94-12.50) | 17.62  (7.01-29.17) | 13.08  (4.76-29.00) | 27.07  (9.41-38.60) | 0.054 |
| NFL | 18.63  (11.83-52.10) | 47.75  (27.83-108.66) | 23.91  (21.40-89.80) | 43.82  (28.86-82.09) | 0.513 |
| NSE | 17.72  (13.07-29.49) | 20.34  (15.95-34.38) | 21.58  (19.23-24.40) | 23.58  (20.31-39.52) | 0.113 |
| S100B | 0.20  (0.13-0.48) | 0.34  (0.21-0.68) | 0.46  (0.11-0.67) | 0.27  (0.20-0.51) | 0.873 |
| Tau | 4.31  (2.20-7.85) | 7.27  (4.68-12.33) | 7.17  (3.92-15.04) | 8.90  (3.33-17.07) | 0.262 |
| UCH-L1 | 123.48  (74.93-378.26) | 251.14  (183.10-477.28) | 360.61  (129.63-528.39) | 466.39  (169.42-899.86) | 0.113 |
| **Sub-cohort with moderate-severe TBI** |  |  |  |  |  |
| Number of patients | 6 (20%) | 3 (10%) | 4 (13%) | 17 (57%) |  |
| Sample time in hours | 11 (10-16) | 14 (12-17) | 21 (17-22) | 16 (10-20) | 1.000 |
| GFAP | 3.32  (2.80-10.12) | 4.46  (2.65-15.19) | 13.19  (5.27-34.00) | 30.31  (18.90-40.99) | **0.036** |
| NFL | 22.77  (12.77-42.35) | 47.75  (27.61-81.74) | 29.98  (21.31-57.16) | 47.21  (37.55-81.19) | 0.168 |
| NSE | 15.14  (12.91-23.07) | 34.10  (24.31-40.44) | 17.74  (14.01-20.90) | 31.91  (22.98-46.36) | **0.036** |
| S100B | 0.18  (0.11-0.22) | 0.24  (0.16-0.49) | 0.07  (0.06-0.27) | 0.29  (0.22-0.51) | 0.155 |
| Tau | 3.50  (2.61-7.77) | 4.70  (2.96-5.98) | 3.43  (2.15-8.04) | 14.36  (4.47-21.49) | 0.054 |
| UCH-L1 | 120.54  (86.19-227.74) | 197.84  (114.92-211.01) | 182.99  (102.86-371.85) | 696.42  (218.56-953.05) | **0.036** |
| **Sub-cohort with unrecorded GCS** |  |  |  |  |  |
| Number of patients | 5 (14%) | 8 (23%) | 6 (17%) | 16 (46%) |  |
| Sample time in hours | 16 (10-20) | 18 (14-20) | 13 (10-20) | 17 (10-18) | 0.893 |
| GFAP | 3.10  (1.20-12.69) | 18.38  (9.17-47.89) | 13.08  (6.08-24.70) | 21.84  (9.39-33.22) | 0.893 |
| NFL | 17.57  (12.26-57.00) | 55.00  (28.14-113.74) | 23.62  (21.40-228.18) | 35.74  (18.39-83.29) | 0.893 |
| NSE | 23.29  (17.72-33.94) | 19.68  (16.44-24.34) | 24.33  (21.29-43.69) | 22.84  (19.34-26.29) | 0.893 |
| S100B | 0.46  (0.18-0.49) | 0.36  (0.22-0.66) | 0.56  (0.46-0.67) | 0.25  (0.16-0.47) | 0.893 |
| Tau | 4.85  (0.97-6.78) | 9.21 (  4.95-15.57) | 10.59  (6.53-21.04) | 4.81  (2.94-11.34) | 0.893 |
| UCH-L1 | 188.37  (56.86-494.04) | 358.82  (230.44-578.85) | 463.33  (255.30-536.75) | 273.19  (128.58-503.10) | 0.893 |

***Supplemental table 4.*** ***Association between serum protein concentration and Adams-Gentry stage.*** *Adams-Gentry stages reflect the severity of axonal injury and have previously been shown to be associated with prognosis. Stage 0 = no foci, Stage 1 = foci in hemispheres only, Stage 2 = foci involving the corpus callosum, Stage 3 = foci involving the brainstem. Values are presented as count (percent) or median (first quartile – third quartile). Serum protein concentrations are measured in ng/ml for GFAP, NSE and S100B, and in pg/ml for NFL, Tau and UCH-L1. Adj. p-value = P-values from the Mann-Whitney U-test adjusted for multiple comparisons. Significant p-values are in bold. TBI = traumatic brain injury, GCS = Glasgow Coma Scale score*

|  |  |  |  |  | **Costs in the United Kingdom (GBP per patient)** | | | **Costs in the United States (USD per patient)** | | |
| --- | --- | --- | --- | --- | --- | --- | --- | --- | --- | --- |
| **Protein** | **Threshold** | **Sensitivity** | **Specificity** | **Patients above threshold** | **MRI for all** | **Protein plus selected MRI** | **Savings** | **MRI for all** | **Protein plus selected MRI** | **Savings** |
| GFAP | 0.63 | 1.00 | 0.08 | 29 (97%) | 385.80 | 389.76 | -3.96 | 2758.56 | 2731.77 | 26.79 |
| NFL | 18.60 | 1.00 | 0.23 | 27 (90%) | 385.80 | 369.67 | 16.13 | 2758.56 | - | - |
| NSE | 16.61 | 1.00 | 0.46 | 24 (80%) | 385.80 | 322.32 | 63.48 | 2758.56 | - | - |
| S100B | 0.06 | 1.00 | 0.15 | 28 (93%) | 385.80 | 381.64 | 4.16 | 2758.56 | - | - |
| Tau | 2.34 | 1.00 | 0.31 | 26 (87%) | 385.80 | 355.42 | 30.38 | 2758.56 | - | - |
| UCH-L1 | 99.09 | 1.00 | 0.31 | 26 (87%) | 385.80 | 350.39 | 35.41 | 2758.56 | 2455.91 | 302.65 |

***Supplemental table 5.*** ***Using serum protein biomarkers for MRI triage in patients with moderate-severe traumatic brain injury.*** *N = 30. Threshold = optimal cut-off for the serum protein concentration measured in ng/ml for GFAP, NSE and S100B, and in pg/ml for NFL, Tau and UCH-L1. “Optimal” means maximising the specificity whilst achieving a minimum sensitivity of 1. MRI for all = cost per patient of subjecting all patients to magnetic resonance imaging (MRI). Protein plus selected MRI = cost per patient of sampling serum protein biomarkers from all patients and taking only those patients for MRI who exceed the threshold serum concentration. Savings = Cost savings when using Protein plus selected MRI compared to MRI for all. To gain an estimate of real-world clinical costs, the United States costs are not based on the assays used in the present study (which are for research only) but on the FDA-approved i-stat platform by Abbott. This platform only measures GFAP and UCH-L1, so costs for other proteins were not calculated.*

| **Protein** | **Feature** | **No BSI** | **BSI without this feature** | **BSI with this feature** | **Adj. p-value** |
| --- | --- | --- | --- | --- | --- |
| GFAP | bilateral | 4.46 (3.11-25.91) | 29.37 (19.91-37.86) | 34.34 (16.31-41.30) | **0.033** |
|  | dorsal | 4.46 (3.11-25.91) | 28.46 (18.90-34.88) | 37.52 (20.00-47.37) | **0.032** |
|  | pontine | 4.46 (3.11-25.91) | 32.51 (21.91-41.59) | 24.08 (8.54-34.34) | 0.067 |
|  | Duret/cont. | 4.46 (3.11-25.91) | 29.37 (11.13-37.86) | 40.99 (31.96-41.30) | **0.032** |
| NFL | bilateral | 27.82 (18.63-47.75) | 45.48 (35.73-58.53) | 65.75 (38.84-114.98) | 0.130 |
|  | dorsal | 27.82 (18.63-47.75) | 39.92 (34.33-47.21) | 73.06 (48.34-159.76) | 0.122 |
|  | pontine | 27.82 (18.63-47.75) | 45.48 (38.52-68.23) | 65.75 (37.55-81.19) | 0.130 |
|  | Duret/cont. | 27.82 (18.63-47.75) | 45.48 (38.14-64.57) | 148.77 (91.55-170.75) | 0.130 |
| NSE | bilateral | 17.14 (13.38-25.05) | 39.49 (24.56-46.16) | 23.18 (18.73-40.05) | 0.059 |
|  | dorsal | 17.14 (13.38-25.05) | 44.45 (23.50-46.36) | 27.20 (20.23-38.36) | 0.059 |
|  | pontine | 17.14 (13.38-25.05) | 39.49 (26.67-46.74) | 20.57 (16.89-23.18) | 0.087 |
|  | Duret/cont. | 17.14 (13.38-25.05) | 29.75 (21.17-45.88) | 45.56 (34.37-83.33) | **0.020** |
| S100B | bilateral | 0.17 (0.07-0.24) | 0.36 (0.23-0.58) | 0.25 (0.22-0.37) | 0.215 |
|  | dorsal | 0.17 (0.07-0.24) | 0.38 (0.27-0.60) | 0.24 (0.19-0.33) | 0.234 |
|  | pontine | 0.17 (0.07-0.24) | 0.36 (0.24-0.53) | 0.23 (0.21-0.29) | 0.215 |
|  | Duret/cont. | 0.17 (0.07-0.24) | 0.30 (0.21-0.50) | 0.29 (0.27-0.44) | 0.207 |
| Tau | bilateral | 4.31 (2.17-7.27) | 20.04 (9.28-42.09) | 11.64 (3.94-15.71) | 0.094 |
|  | dorsal | 4.31 (2.17-7.27) | 19.67 (8.90-21.49) | 12.93 (4.20-18.32) | 0.090 |
|  | pontine | 4.31 (2.17-7.27) | 20.04 (10.03-28.78) | 4.47 (3.41-11.64) | 0.103 |
|  | Duret/cont. | 4.31 (2.17-7.27) | 11.01 (3.67-21.92) | 17.07 (15.71-18.74) | 0.056 |
| UCH.L1 | bilateral | 123.48 (98.13-262.49) | 732.60 (418.99-999.10) | 696.42 (193.99-865.64) | **0.031** |
|  | dorsal | 123.48 (98.13-262.49) | 596.43 (390.59-1014.45) | 737.49 (197.23-912.47) | **0.031** |
|  | pontine | 123.48 (98.13-262.49) | 791.63 (475.80-968.40) | 218.56 (169.42-780.99) | **0.031** |
|  | Duret/cont. | 123.48 (98.13-262.49) | 682.50 (181.71-952.36) | 696.42 (600.31-849.25) | **0.020** |

***Supplemental table 6. Association of protein concentration with adverse features of axonal brainstem injury in the moderate-severe sub-cohort.*** *BSI = brainstem injury. The table lists the serum concentration of biomarkers as median (interquartile range). Whether there was a trend in the concentration from no BSI to BSI without the adverse feature to BSI with the adverse feature, was assessed with a two-sided Jonkheere-Terpstra test and p-values adjusted using a false discovery rate threshold of 5%. Significant p-values are in bold.* *Duret/cont. = Duret haemorrhage or contusion*

# References

1. NHS England. National Schedule of NHS Costs FY1920. https://www.england.nhs.uk/costing-in-the-nhs/national-cost-collection/

2. British Medical Association. Pay scales for junior doctors in England. https://www.bma.org.uk/pay-and-contracts/pay/junior-doctors-pay-scales/pay-scales-for-junior-doctors-in-england

3. NHS Employers. HCAS pay scales 2021/22. Accessed 03 Aug 2022, https://www.nhsemployers.org/articles/hcas-pay-scales-202122

4. New Choice Health I. Brain MRI Cost and Procedure Information. Accessed 17 Aug 2022, https://www.newchoicehealth.com/procedures/brain-mri

5. salary.com. Hourly Wage for Medical Resident Salary in the United States. Accessed 17 Aug 2022, https://www.salary.com/research/salary/posting/medical-resident-hourly-wages

6. U.S. Bureau of Labour Statistics. Occupational Employment and Wage Statistics. Accessed 17 Aug 2022, https://www.bls.gov/oes/current/oes291141.htm
